# Substance-Induced Psychiatric Disorders, Epigenetic and Microbiome Alterations, and Potential for Therapeutic Interventions

**DOI:** 10.3390/brainsci14080769

**Published:** 2024-07-30

**Authors:** Shabnam Nohesara, Hamid Mostafavi Abdolmaleky, Sam Thiagalingam

**Affiliations:** 1 Department of Medicine (Biomedical Genetics), Boston University Chobanian and Avedisian School of Medicine, Boston, MA 02118, USA; sabdolma@bidmc.harvard.edu; 2 Mental Health Research Center, Psychosocial Health Research Institute, Department of Psychiatry, School of Medicine, Iran University of Medical Sciences, Tehran 14535, Iran; 3 Nutrition/Metabolism Laboratory, Department of Surgery, BIDMC, Harvard Medical School, Boston, MA 02215, USA; 4 Department of Pathology & Laboratory Medicine, Boston University Chobanian and Avedisian School of Medicine, Boston, MA 02118, USA

**Keywords:** substance use disorder, psychotic symptoms, epigenetic alterations, substance use disorder–gut microbiome interactions

## Abstract

Substance use disorders (SUDs) are complex biopsychosocial diseases that cause neurocognitive deficits and neurological impairments by altering the gene expression in reward-related brain areas. Repeated drug use gives rise to alterations in DNA methylation, histone modifications, and the expression of microRNAs in several brain areas that may be associated with the development of psychotic symptoms. The first section of this review discusses how substance use contributes to the development of psychotic symptoms via epigenetic alterations. Then, we present more evidence about the link between SUDs and brain epigenetic alterations. The next section presents associations between paternal and maternal exposure to substances and epigenetic alterations in the brains of offspring and the role of maternal diet in preventing substance-induced neurological impairments. Then, we introduce potential therapeutic agents/approaches such as methyl-rich diets to modify epigenetic alterations for alleviating psychotic symptoms or depression in SUDs. Next, we discuss how substance use–gut microbiome interactions contribute to the development of neurological impairments through epigenetic alterations and how gut microbiome-derived metabolites may become new therapeutics for normalizing epigenetic aberrations. Finally, we address possible challenges and future perspectives for alleviating psychotic symptoms and depression in patients with SUDs by modulating diets, the epigenome, and gut microbiome.

## 1. Introduction

Substance use disorder (SUD) is defined as a chronic state of the uncontrolled exploration and use of drugs that exert detrimental effects on the family, society, and professional aspects of a patient’s life. The high prevalence of psychotic symptoms such as hallucinations and delusions has been reported in patients with substance use [1]. For instance, cannabis and amphetamine users exhibit a higher prevalence of psychotic disorders and cognitive symptoms like schizophrenia [2,3,4]. Moreover, a greater frequency of both opioid and cocaine use has been reported in individuals with psychotic symptoms compared to individuals with nonpsychotic symptoms [5]. Similar to other neuropsychiatric diseases, the etiology of SUDs is complex and multifactorial, in which a variety of responsible genes interplay among each other and with the environment. This interplay alerts neuronal function and structure in different brain areas and gives rise to continuous changes at the cellular, molecular, and behavioral levels [6]. For example, it appears that amphetamine affects brain function via interplay with nerve terminals that utilize indoleamines, like serotonin and catecholamines, including norepinephrine and dopamine as multifunctional neurotransmitters [7]. The prevalence of psychotic symptoms in substance users may be due to the burst release of dopamine in the striatum and subsequently excessive secretion of glutamate into the brain cortex, which further leads to the injury of cortical interneurons and the disruption of thalamocortical signals [8]. It has been found that there is also a potent relationship between any type of SUD and the polygenic risk score for schizophrenia [9]. Genetic factors with a heritability of almost 50% (*h*^2^ = ~50%) are associated with SUDs and their adverse consequences. For example, alcohol-related tendencies can be affected by loci in alcohol-metabolizing genes (e.g., *ADH1B* and *ALDH2*), and nicotine-related tendencies can be influenced by loci within the *CHRNA5–CHRNA3–CHRNB4* gene cluster [10]. More information about the genetics of SUDs is provided in a review written by Gelernter and Polimanti [11].

**Figure 1:**
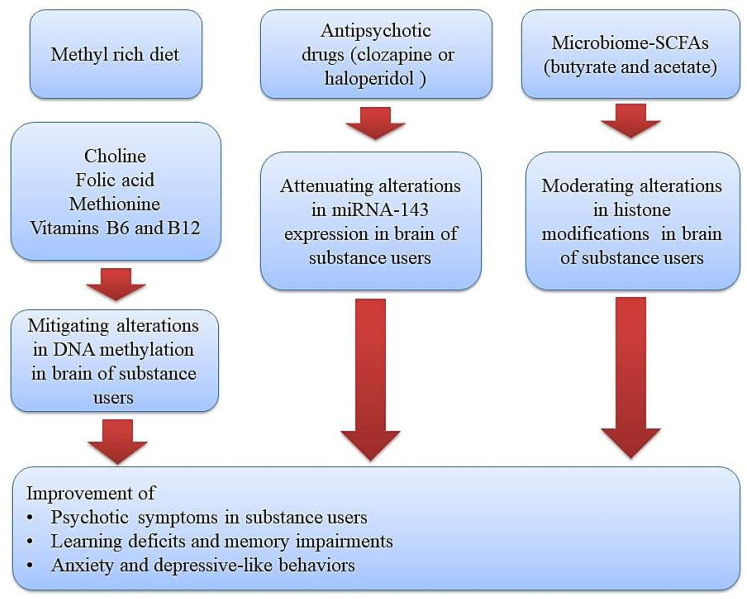
Therapeutic approaches using diet or epigenetic drugs (methyl donor micronutrients, antipsychotic drugs, and gut microbiome-derived metabolites) for improving substance-induced neurological impairments via normalizing epigenetic aberrations. Methyl donor nutrients, such as methionine, choline, folate, and some B vitamins, participate in one-carbon metabolism and hence could serve as potential epigenetic diets to reduce substance-induced neurological impairments. Likewise, antipsychotic drugs and gut microbiome–derived metabolites like butyrate and acetate can target a gene for epigenetic regulation and thus could serve as potential epigenetic modifiers to improve psychotic symptoms, learning and memory impairments, and depressive-like behaviors in substance users.

The tight relationship among genes and environmental factors in the development of psychotic symptoms in substance users can be mediated by epigenetic mechanisms as well [12]. During this process, transcription factors and some specific enzymatic protein complexes play a critical role in modulating gene expression and creating long-lasting alterations through modification of chromatin structure [13,14]. These chromatin-modifying mechanisms or other epigenetic alterations have the capacity to alter gene expression without changing DNA sequences. Moreover, drug use or the toxic effects of alcohol may create disturbances in the absorption of micronutrients (omega–3, choline, vitamins, and folic acid) and hence imbalances in the levels of methyl donors, which further give rise to the development of neuropsychiatric diseases via brain epigenetic changes, especially DNA methylation [15]. Therefore, diet modifications and using supplementations with adequate levels of methyl donors is a promising strategy in alleviating the development of psychotic symptoms and neurological impairments in substance users (Figure 1).

This narrative review aims to elaborate links between substance use-induced neuropsychiatric impairments and epigenetic alterations in brain tissue and the role of diet modifications in alleviating such deficits via normalizing epigenetic aberrations. To this end, we briefly present associations between substance use and the development of psychotic symptoms and neuropsychiatric diseases via epigenetic aberrations. We will discuss studies that support the link between substance use and epigenetic alterations, including DNA methylation, histone modifications, and microRNAs (miRNAs), in particular, in the brain tissue. Note that in DNA methylation, a methyl group is added to a cytosine residue, or less frequently, to an adenine residue that is followed by guanine. This process, mediated by different enzymes, results in methylated cytosines acting as targets for DNA-binding proteins (e.g., MeCP2, MBD1, MBD3, and MBD4), which mediate chromatin condensation and gene silencing. Histone modifications are another type of epigenetic regulation, during which different amino acids of histone tail proteins can become acetylated or methylated (mediated by various enzymes). These modifications affect the positive electric charge of histone proteins and thus the intensity of their binding to DNA, which has a negative electric charge. Histone acetylation generally decreases chromatin condensation and stimulates gene expression, whereas histone methylation can either increase or decrease gene expression depending on the identity or location of the methylated amino acids of histone tail proteins. Additionally, in RNA interference, another type of epigenetic regulation, miRNAs—small non-coding RNAs approximately 20 bases in length—bind to their cognate RNAs and inhibit gene transcription or promote RNA degradation [16].

In this work, we will summarize those studies that show the impact of paternal and maternal exposure to substances on epigenetic alterations in the brains of offspring and the role of maternal diet on the prevention of substance induced neurological impairments notably psychosis, possibly via epigenetic mechanisms. In addition, we provide an overview on the use of different types of diet, especially the methyl-rich diet, for alleviating psychotic symptoms and depressive-like behaviors in patients with SUDs. The next step involves determining how substance use–gut microbiome interactions contribute to the development of psychotic symptoms and depressive-like behaviors through epigenetic alterations and how gut microbiome-derived metabolites help researchers in the design of new therapies based on normalizing epigenetic aberrations. The last section discusses potential challenges and presents future perspectives relevant to alleviating psychotic symptoms and depressive-like behaviors in patients with SUDs using diet modifications and modulation of the gut microbiome.

## 2. Association between Substance Use and the Development of Psychotic Symptoms and Depressive-Like Behaviors

There is increasing evidence that drug use is associated with the development of psychotic symptoms in various contexts encompassing substance withdrawal, acute or chronic intoxication, in the form of substance-induced psychosis, and delirium [17]. Substance-induced psychosis is described as a condition in which the onset of psychosis appears to be due to substance use, but it remains for days, weeks, or even months in the absence of substance use [18]. Long-term neuropsychiatric deficits induced by substance use are mainly attributed to the activation of different signaling pathways relevant to triggering and the progression of oxidative stress and inflammation [19,20]. For example, methamphetamine-induced psychosis is related to changes in the balance of the immune system, the activation of a variety of chemokines and cytokines (e.g., IL-1α, CCL11, and CCL27), elevated lipid peroxidation, and decreased antioxidant defenses [21,22]. Persistent psychotic symptoms can be induced by amphetamines, cannabis, and alcohol [23]. It is estimated that almost 40% of methamphetamine users suffer from psychotic symptoms like hallucinations and delusions in addition to violence, impulsivity, and cognitive disturbance [24,25].

Mechanistically, the emergence of psychotic symptoms in patients with SUDs could be linked to epigenetic alterations leading to gene expression dysregulation. For example, it was found that decreased DNA methylation at a particular dopamine receptor type 4 (DRD4) CpG2.3 unit was associated with paranoid symptoms in patients with methamphetamine use disorder, and higher methylation levels at the catechol-O-methyltransferase (COMT) CpG 51.52 unit was linked to reduced motor-impulsivity scores in the same set of patients [26]. In another study, Veerasakul et al. found a significant elevation in parvalbumin (PVALB) DNA methylation in methamphetamine-induced psychosis, indicating that methamphetamine dependence confers the GABAergic deficits by epigenetic changes [27]. Kalayasiri et al. reported a strong link between methamphetamine-induced paranoia and alterations in long interspersed element-1 methylation patterns, which modulate the immune and neuro-oxidative pathways [28]. In addition to DNA methylation and histone modifications, substance-induced psychosis is connected to alterations in miRNAs. In this line, an interesting study demonstrated that patients with methamphetamine-induced psychosis exhibited significant differences in the levels of miR-let-7d, miR-let-7e, miR-15b, and miR-181a compared to control subjects [29]. In a more recent study, Chen et al. reported that psychological comorbidities in substance users are linked to the dysregulation of some crucial exosomal miRNAs connected to changes in the levels of certain neurotransmitters, such as serotonin, in these patients [30]. In another study by the same group, they found a negative correlation between the expression levels of exosomal miR-92a-3p, miR-16-5p, miR-129-5p, and miR-363-3p and Hamilton Anxiety/Depression scores in methamphetamine-dependent patients as well as heroin-dependent patients.

In the following sections, we address more details pertaining to the link between substance use and substance-induced psychiatric diseases. Overall, current findings demonstrate that substance use is associated with the development of psychotic symptoms and depressive-like behaviors, and such malfunctions are mediated by epigenetic shifts causing gene expression dysregulation.

## 3. The Effects of Substance Use on Changing Brain Functions via Epigenetic Alterations

**Figure 2:**
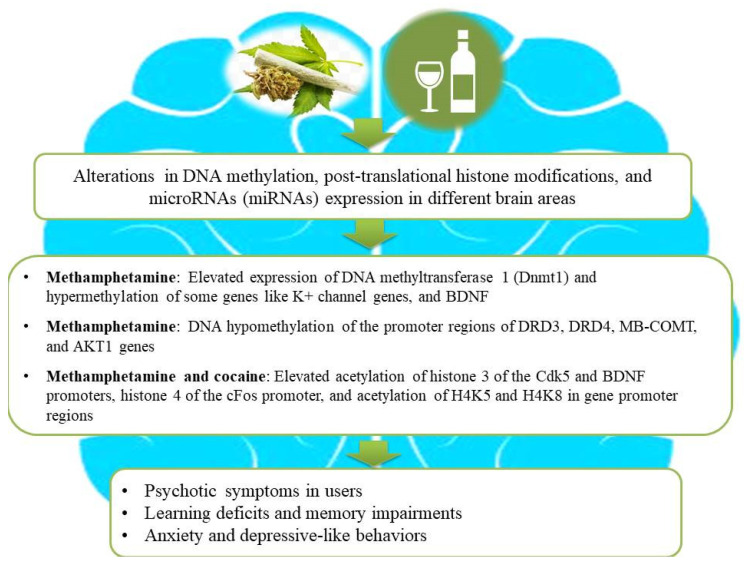
An illustration of association among substance use, epigenetic aberrations, and the development of neurological impairments in users. Substance use causes aberrant alterations in DNA methylation, post-translational histone modifications, and microRNA (miRNA) expression in different brain areas of substance users and subsequently gives rise to the development of psychosis, depressive-like behaviors, and learning or memory deficits. Ethanol intake and illicit drugs (for example, cannabis, methamphetamine, and cocaine) lead to changes in gene expression by combinatorial epigenetic events and, hence, increase the risk of developing psychosis and other neurological impairments.

Epigenetic regulatory mechanisms such as DNA methylation, histone modifications, and miRNAs play powerful roles in adaptive alterations in neuroplasticity following prolonged drug use [31,32]. Previous studies have shown that neuronal functions relevant to learning, memory, and synaptic plasticity can be dynamically regulated by DNA methylation and histone modifications in individuals with SUDs [33,34]. For example, heroin-induced remodeling of the actin cytoskeleton via alterations in DNA methylation levels might participate in behavioral plasticity [35]. In this process, the proteasomal degradation of DNA methyltransferase DNMT3a by the E2 ubiquitin-conjugating enzyme contributes to the initiation of CaMKK1 gene transcription and the elevation of CaMKK1 protein expression via decreasing DNA methylation of its promoter region and thereby facilitates actin polymerization through the activation of the CaMKIα/βPIX/Rac1 pathway in the dorsal hippocampus [35]. Likewise, there is an interesting association between DNA hypermethylation of the dopamine transporter gene (DAT1) and dopamine release in individuals addicted to psychoactive substances [36]. Furthermore, reduced levels of brain-derived neurotrophic factor (BDNF) methylation in CpG 5–11 have been found in subjects with tobacco use and depression compared to those who did not consume tobacco with or without depression [37]. In addition to DNA methylation, histone modifications may play important roles in altering neuronal functions in subjects with SUDs [38]. For example, human primary astrocytes treated with opioid and psychostimulants exhibited elevated levels of global acetylation of H3 histone lysine residues, except for the acetylation of the 14th lysine residue [39]. It is hypothesized that illicit drugs like ∆9-tetrahydrocannabinol have the capacity for the activation of histone deacetylases, causing heterochromatic sequences of genes involved in cognitive functions, and higher risks of schizophrenia development and aggravation [40]. Similarly, the retrieval of heroin-related memories is associated with changes in histone acetylation during reconsolidation, and hence interventions to modulate histone acetylation can serve as valid approaches to cure SUD and hamper relapses [41]. Another study showed that adolescent-intermittent ethanol exposure diminishes the level of H3 acetylation in the hippocampus, reduces the expression of BDNF, and, subsequently, suppresses neurogenesis in this brain region [42]. Figure 2 illustrates how drug use and alcohol consumption increase the risk of psychosis, anxiety, depressive-like behaviors, and learning and memory deficits in users via epigenetic alterations.

Other lines of evidence linking substance use to epigenetic alterations, including DNA methylation and histone modifications in the different brain areas, are summarized in Table 1 and Table 2, respectively. In addition to DNA methylation and histone modifications, substance use epigenetically alters gene expression by changing the levels of endogenous non-coding RNAs, like miRNA and circular RNA (circRNA), in the brain tissue [43,44,45]. circRNAs are capable of influencing substance behavioral effects through interplay with miRNAs [44]. For instance, striatal miRNAs play powerful roles in neuroplasticity, learning and memory, and reward circuit function and regulation [46,47]. As another example, a study by Chavoshi et al. indicated that astrocyte over-activation and striatal atrophy following treatment with methamphetamine is connected to 167 differentially expressed miRNAs in the striatum region [48]. Gu et al. reported that heroin users could exhibit elevated serum levels of miR-486-5p, miR-206, and let-7b-5p, whereas methamphetamine users had increased serum levels of miR-9-3p [49]. Another study found that elevated levels of let-7b-3p in the nucleus accumbens and the ventral tegmental area of methamphetamine users may be considered a potential biomarker for the diagnosis of addiction in these patients [50]. Furthermore, miRNA–mRNA network analysis of postmortem brains and blood samples of subjects with opioid use disorder revealed a potent overlap between their differentially expressed target genes, despite the distinct profiles of the altered brain and blood miRNAs [51].

More studies linking substance use to the changes in the expression of miRNAs in the brain tissue are summarized in Table 3.

Generally, aberrant changes in DNA methylation, post-translational histone modifications, and miRNA expression in various brain regions of substance users heavily affect neuronal functions relevant to synaptic plasticity, learning, and memory, which may accelerate the development of psychiatric disorders. However, it is crucial to recognize that while these epigenetic alterations are associated with substance use, the exact causal mechanisms and their implications for psychiatric disorders require further investigation. Understanding these complex interactions will necessitate extensive research to differentiate between correlation and causation and develop targeted therapeutic interventions.

## 4. Paternal and Maternal Substance Use and Epigenetic Alterations in the Brains of the Offspring

**Figure 3:**
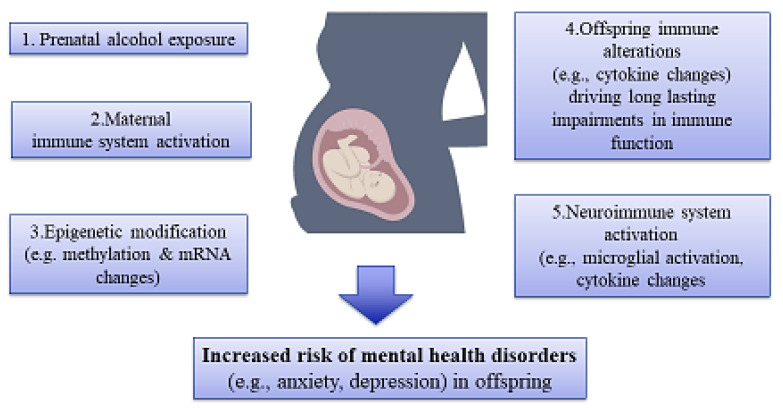
An illustration of association among maternal substance use, epigenetic alterations, and risk of mental disorders in offspring. Prenatal exposure to substances like alcohol affects the immune system, epigenetic mechanisms, and, hence, the development of mental disorders in offspring.

It has been reported that illicit drugs and other substances are capable of passing via the placenta, activation of the immune system, and disrupting the development of offspring by changing gene expression and/or causing epigenetic aberrations in various body organs, especially the brain tissue, and subsequently increasing the risk of mental disorders (Figure 3) [110,111].

For example, methamphetamine has been found to be the most common illicit drug taken by pregnant mothers, which gives rise to changes in the expression of neurodevelopment-related genes and thereby cognitive deficits and neuropsychiatric diseases in offspring [112,113]. Other lines of evidence linking paternal and maternal substance use during pregnancy to epigenetic changes with respect to DNA methylation and histone modifications in various brain regions are summarized in Table 4. It has been found that maternal substance use during pregnancy is connected to an elevated risk of psychotic symptoms in offspring as well [114]. For example, both paternal and maternal cannabis use is linked to the higher number of psychotic-like experiences in the offspring in childhood (at age ten years) [115]. The development of psychotic symptoms and other neurological impairments in offspring owing to substance use can be mediated by epigenetic changes. For instance, a recent study by Wendt Viola et al. indicated that prenatal cocaine exposure in humans can increase the risk for psychosis in offspring, which is connected to epigenetic alterations [116]. In another study by Hollins et al., it was shown that a combination of prenatal treatment with poly I:C and cannabinoid exposure caused strong differences (98%) in the miRNA expression of the brain hemispheric region within the Dlk1-Dio3-imprinted domain on 6q32 that is related to the syntenic human locus in schizophrenia [117]. A more recent genome-wide human study showed that subjects with cannabis use disorder had differential DNA methylation at four CpG sites, remarkably at the AHRR cg0557592 site, found to be an important mediator linking cannabis use to mental disorders, particularly mood disorders [118]. Moreover, maternal exposure to e-cigarette aerosols with nicotine could impair short-term memory, which was connected to the elevated levels of global DNA methylation in the brains of offspring [119].

There is evidence showing that such epigenetic changes in offspring can occur due to malabsorption of nutrients in pregnant women with SUDs. In this line, it has been shown that drug use and alcohol consumption result in derangements in the absorption of micronutrients such as folic acid, choline, and omega 3 during pregnancy, and hence supplementation with such diets in pregnant women with SUD may prevent neurological deficits in the offspring through normalizing epigenetic aberrations [120,121,122,123]. As another interesting example, it was shown that prenatal alcohol exposure caused perturbances in hippocampal miRNA expression, and choline supplementation could reverse an ethanol-dependent increase in hippocampal miR-200c expression [124]. Another animal study demonstrated that alcohol consumption increases DNA methylation in the PFC and hippocampus of rat pups during the neonatal period, and supplementation with choline could reduce DNA hypermethylation in both of these brain regions [125]. Collectively, these findings show that drugs and other substances like alcohol can pass through the placenta, activate the immune system, impair the development of offspring, and elevate the risk of mental illnesses by altering gene expression and/or causing epigenetic aberrations in brain tissue. However, while these studies provide significant insights, further research is needed to fully understand the mechanisms involved and determine the short- and long-term effects of these substances on human development and mental health.

Table 4 shows more examples of the associations between paternal and maternal substance use and epigenetic alterations in various brain regions in offspring.

**Table 4:** Paternal and maternal substance use and epigenetic changes in various brain regions of offspring.

Type of Substance/Type of Study	Brain Area	Epigenetic Changes	Effects on the Brains of Offspring	Ref.
Long-term parental methamphetamine exposure in mice	Hippocampus	DNA methylation	Presence of DMSs in the brains of offspring exposed to methamphetamine during embryonic development compared to control	[126]
Maternal methamphetamine exposure in mice	NA	DNA methylation	DMSs of some genes involved in neurodevelopmental process	[127]
Maternal cocaine exposure in mice	Hippocampus	DNA methylation	Reducing global DNA methylation at 3 and elevation of global DNA methylation at 30 days postpartum	[128]
Prenatal cocaine exposure in mice	Hippocampus	DNA methylation	Cognitive deficits in offspring due to overexpression of DNMT 1 and L-methionine and subsequently elevating DNA methylation of IGF-2	[129]
Maternal alcohol consumption	Hippocampus	DNA methylation	Alterations in DNA methylation, gene expression, and brain function in offspring	[130]
Maternal alcohol consumption	Hippocampus	DNA methylation	Elevated levels of *dnmt1*, *dnmt3a*, and *hdac2* in offspring	[131]
Paternal cocaine exposure in rats	Hippocampus	Histone acetylation/methylation	Elevated levels of a single methylated lysine 4 on histone H3 (H3K4me1) and acetylated histone H3 (H3Ac) near the Dao1 gene responsible for the oxidative deamination of D-serine (an amino acid with antipsychotic activity)	[132]
Prenatal cocaine exposure in mice	Frontal cortex	Histone acetylation	Hyperacetylation of histone H3 at the *BDNF* promoter and thereby elevating the mRNA and protein levels of *BDNF* at adult postnatal day 60	[133]
Parental morphine exposure in rats	PFC and hippocampus.	Histone acetylation	Decrease in histone H3 acetylation and ΔFosB in the offspring of morphine-withdrawn parents during postnatal days 5, 21, 30, and 60	[134]
Prenatal morphine exposure in rats	VTA	Histone acetylation	Overexpression of HDAC5	[135]
Maternal cannabis exposure	NA	Histone lysine methylation	Reduced 3meH3K4 and elevated 2meH3K9 repressive marks at the DRD2 gene locus in the cannabis-exposed offspring	[136]
Perinatal alcohol and nicotine–alcohol exposure in rats	VTA	miRNA	Alterations in the expression of miRNAs in dopaminergic neurons	[137]
Prenatal alcohol exposure in mice	Brain cortex and microvascular endothelium at embryonic day 18	miRNA	Elevated levels of MicroRNA-150-5p and suppressing the angiogenic factor Vezf1	[138]

## 5. Therapeutic Approaches Using Diet Modification or Epigenetic Drugs to Improve Psychotic Symptoms, Learning Deficits, and Memory Impairments in Animal Models and Patients with SUDs

Several lines of evidence have indicated that psychotic symptoms in patients with SUDs are associated with changes in the nutrients that influence methylation machinery for post-transcriptional gene regulation. For example, lower levels of folate, a cofactor for methylation reactions involved in gene transcription regulation levels [139], have been reported in psychotic methamphetamine users versus non-psychotic methamphetamine users. In fact, every 1-unit decrease in serum folate level may increase the risk of psychosis by 27% [140]. Therefore, the use of a methyl-rich diet is considered a promising strategy for alleviating psychotic symptoms in patients with methamphetamine use. As another remarkable example, one study showed DNA hypomethylation of the promoter regions of the *DRD3*, *DRD4*, *MB-COMT*, and *AKT1* genes in patients with methamphetamine psychosis [141]. It was concluded that the use of a methyl-rich diet may help improve psychotic symptoms in these patients [141]. Tian et al. also reported that repeated treatment with methionine (the main methyl donor amino acid in mammals) for 25 days before and during conditioned place preference training could suppress the establishment of cocaine rewarding effects by reversing global DNA hypomethylation in the PFCs of mice [142].

In another study, Wright et al. found that global hypomethylation and decreased methylation at CpG dinucleotides in the c-Fos gene promoter are connected to cocaine-induced c-Fos expression in the nucleus accumbens cores of rats [143]. Their results revealed that prolonged methyl supplementation by L-methionine could alleviate drug-seeking attitudes and behavioral sensitization to the locomotor-activating effects of cocaine by enhancing DNA methylation of the c-Fos promoter region [143]. Likewise, treatment with choline (the main source of methyl groups in mammals) is capable of reversing detrimental effects of alcohol on brain function [121]. As other examples, in the adult offspring of rats who consumed alcohol during pregnancy, elevated levels of MeCP2 (methyl-CpG-binding protein), the Dnmt1 enzyme (DNA-methylating enzyme 1), and several repressive histone marks (Setdb1, H3K9me2, and G9a), along with reduced levels of histone activation marks (H3K9ac, H3K4me3, and H3S10 phosphorylation), were reported in the thalamic β-EP-producing proopiomelanocortin neurons, which were normalized by gestational choline supplementation [144]. Gitik et al. also identified 462 genes with altered promoter DNA methylation in adult mice dorsal hippocampus after nicotine exposure associated with learning deficits. They found that dietary choline supplementation was capable of reducing learning deficits in mice exposed to nicotine by normalizing DNA methylation of the hippocampus [145]. In addition to methyl-rich diets, a ketogenic diet is a promising candidate for reducing neurotoxicity in substance users. During alcohol detoxification, a paradoxical energy-deficit state occurs in the human brain owing to reduced plasma levels of acetate and beta-hydroxybutyrate as epigenetic modifiers, which further contributes to withdrawal symptoms and neurotoxicity in patients with alcohol use disorder [146]. Wiers et al. found that a shift in energy substrates during withdrawal in patients with alcohol use disorder may be a major reason for withdrawal severity and neurotoxicity, and a ketogenic diet could contribute to reducing withdrawal symptoms by elevating ketone bodies (beta-hydroxybutyrate, acetoacetate, and acetone) and decreasing levels of neuroinflammatory markers [147]. In another study, the same group reported that three weeks of treatment with a ketogenic diet is capable of reducing a neurobiological craving signature in patients with alcohol use disorder [148].

Likewise, some drugs are capable of maintaining neuronal function in substance users by reversing epigenetic aberrations. For instance, Wang et al. reported that acute treatment of mice with phencyclidine resulted in a drastic decrease in miRNA-143 expression in astrocytes of the PFC region and hence the development of psychotic symptoms resembling schizophrenia. Their results revealed that while a D2 receptor-specific agonist (quinpirole) also decreased miRNA-143 expression, antipsychotic drugs like clozapine or haloperidol could prevent phencyclidine-induced hyperactivity by restoring miR-143 expression and suppress D2 receptors induced expression of Neuregulin-1, a target of miRNA-143 [149]. In another study, it was found that cocaine could impair DNMT activity in astrocytes, which, in turn, accelerates neurodegeneration [150]. However, Piracetam, a drug for the treatment of cognitive disorders, could hamper cocaine induced-impairments of DNMT activity and reduce cell death [150]. In sum, psychotic symptoms, learning deficits, and memory impairments in patients with SUDs are linked to altering the nutrients capable of modulating methylation machinery for post-transcriptional gene regulation, and, hence, methyl donor micronutrients or epigenetic drugs may be considered as potential candidates to prevent or treat such abnormalities in patients with SUDs. However, it is important to note that the results from animal studies presented in this work cannot be directly translated to humans; clinical trials are necessary to validate the efficacy of these approaches and their potential side effects in human subjects.

## 6. Substance Use-Induced Gut Microbiome Alterations May Intensify Psychopathology via Epigenetic Aberrations

Accumulating evidence has demonstrated that substance use contributes to the disturbance of gut barrier integrity, elevation of intestinal permeability, and changing the gut microbiota composition, which further result in derangements in brain function and the patient’s mental status. Alterations in the gut microbiota and their metabolites by substance use heavily affect brain function by causing unfavorable shifts in the immune and inflammatory pathways and the release of specific neurotransmitters [151,152]. Numerous bacteria have demonstrated their ability to produce different types of neurotransmitters, like serotonin, dopamine, and GABA [153]. While the link between gut dysbiosis and the pathogenesis of various psychiatric diseases are reviewed elsewhere [154], the impacts of substance use on the gut microbiome and corresponding neurochemical changes in the brain tissue may also contribute to the development of psychiatric disorders [155]. For instance, as patients with psychiatric disorders exhibit reduced abundance of the butyrate-producing *Faecalibacterium* and increased abundance of pathogenic and pro-inflammatory bacteria such as *Eggerthella* and *Streptococcus*, methamphetamine use can lead to similar bacterial dysbiosis as well [154,156,157].

A growing body of evidence has demonstrated a close relationship between SUDs and gut microbiome dysbiosis [158,159,160]. Opioid-induced bowel dysfunction is one of the adverse effects of chronic opioid use [161]. A clinical study showed that methadone-treated individuals exhibited lower fecal bacterial α-diversity and composition compared to non-opioid users [162]. In addition, patients with heroin use disorder exhibited drastic changes in gut microbiome diversity, composition, and functions, and the abundance levels of *Turicibacter*, *Actinomyces*, and *Weissella* bacteria could be considered biomarkers for predicting heroin-induced depression symptoms [163]. In methamphetamine users, it has been shown that an altered gut microbiome is associated with cognitive decline, psychotic syndrome, and the pathogenesis of methamphetamine-induced psychosis [164]. Furthermore, elevated abundance levels of *Lachnospiraceae*, *Xanthomonadale*, *Romboutsia*, and *Sphingomonadales*, as well as decreased abundance levels of *Bacteroidaceae* and *Deltaproteobacteria*, were connected to the development of psychotic symptoms in methamphetamine users [164]. Interestingly, fecal microbiota transplantation from methamphetamine-administered mice was also capable of creating methamphetamine-induced anxiety- and depressive-like behaviors and elevating neuroinflammation in the hippocampus region of recipient mice [165].

In another study, Panee et al. found that the lower Prevotella–Bacteroides ratio of the fecal microbiome in marijuana users was associated with cognitive deficits [166].

Remarkably, alerted gut microbiome composition in subjects with SUDs can heavily influence the production of gut microbiome-derived metabolites as well, which further affect mental health. For example, a reduction in the abundance of *Akkermansia muciniphila*, a species responsible for the production of some metabolites involved in modulating the expression of tight junction proteins and maintenance of intestinal barrier integrity, was seen in methadone-treated individuals in comparison with non-opioid users [162]. It has been reported that substances like cannabis, nicotine, and methamphetamine have a great impact on the regulation of bacterially derived products like neuroactive metabolites, epigenetic modifiers, neurotransmitters, and anti-inflammatory metabolites, which play critical roles in the cross-talk between the gut and the central nervous system (CNS) [167,168,169,170]. For instance, a reduced concentration of butyric acid, an epigenetic modifier and anti-inflammatory metabolite involved in preventing the development and progression of neuropsychiatric diseases, has been reported as the result of oral and fecal bacteria alterations in patients with cocaine use disorder [171]. Overall, it appears that systemic inflammation in methamphetamine use disorder is due to the decreased abundance levels of butyrate-producing bacteria like *Faecalibacterium*, *Dorea*, and *Blautia* as well as increased abundance of pro-inflammatory bacteria [157,172,173].

Therefore, dietary sodium butyrate supplementation and the microbiome-derived short-chain fatty acids (SCFAs) may act as potent epigenetic modifiers and anti-inflammatory agents to treat drug-induced toxicity and substance-induced psychosis [174,175,176,177]. Some supporting evidence comes from the recent Zhang et al. studies showing that gut microbiome-derived SCFAs could exhibit great potential for reducing methamphetamine-induced anxiety- and depressive-like behaviors by suppressing colonic inflammation and improving gut homeostasis [178]. In another study, it has been shown that sodium butyrate supplementation is capable of alleviating detrimental effects of alcohol use disorder on the CNS by inhibiting neuroinflammation [179]. Some therapeutic agents are also capable of improving mental disorders induced by methamphetamine use through increasing the abundance of butyrate-producing and hydrogen-producing bacteria. For example, Wang et al. reported altered gut microbial composition (decreased abundance of butyrate-producing bacteria like *Bacteroides* and *Roseburia*), reduced alpha diversity, and elevated self-rating scales of depression (SDS) and anxiety (SAS) in methamphetamine users compared to their age-matched healthy subjects [180]. They found that inhaling hydrogen could improve neuropsychiatric impairments induced by methamphetamine use through changing gut microbiota profiles and increasing the abundance of *Bacteroides* and *Roseburia* [180]. Considering current data indicating that SCFAs are affected in SUDs and microbial or other therapeutic interventions improve substance-induced psychiatric symptoms by increasing butyrate or other SCFAs levels, it is conceivable to suggest that epigenetic alterations mediate mental health impacts of substance-induced gut dysbiosis in SUDs. Taken together, these findings indicate that substance use is capable of disturbing the gut barrier’s integrity, increasing intestinal permeability, and alterations in the gut microbiota composition, which further lead to disturbances in brain function and the patient’s mental status. As an example, since gut microbiome dysbiosis in patients with SUDs is related to a reduced abundance of butyrate-producing bacteria and increased abundance of pathogenic and pro-inflammatory bacteria, dietary sodium butyrate supplementation and/or SCFA-producing probiotics may serve as epigenetic remedies to reduce the risk of mental illnesses in substance users pending confirmation in human clinical studies.

## 7. Challenges and Potentials for Clinical Translation

In order to accelerate translational relevance, consistency in substance exposure paradigms and the establishment of human-relevant dosage regimens or change to volitional models of substance exposure are essential. In addition, epigenetic alterations in the brain tissue should be assessed in both short-term and long-term use/exposure or withdrawal to precisely estimate the stability of substance-induced epigenetic aberrations [181]. Moreover, in order to obtain greater insights on shared substance-induced neuroplasticity changes and neurological impairments and promote clinical translation based on these findings, systematic comparisons of different substances and their epigenetic consequences are required. Some studies have shown that there are sex-dependent differences in substance-induced epigenetic modifications in the brain; hence, future studies should have more of a focus on unresolved issues to explore how sex differences affect substance-induced epigenetic alterations [182].

In order to achieve more effective diagnostic and therapeutic strategies for substance-induced neurological impairments, single-cell next-generation sequencing technologies and approaches should be utilized more extensively in future research relevant to substance-induced epigenetic alterations in the brain tissue. Single-cell RNA sequencing can also be applied for the detection of new gene targets regulated by opioids and other substances. Investigation of the genome for areas of opened or closed chromatin after short or long-term exposures to substance can be conducted by ATAC-seq. ChIP-seq is capable of connecting this type of epigenetic alteration with the affected gene loci. Moreover, locus-specific epigenetic editing tools provide an opportunity for researchers to detect the functional consequences of substance-induced epigenetic alterations via manipulating such targets in a cell type-specific manner [183,184].

Owing to the complexity of experimental variables like the host’s genotype and diet and the difficulty of controlling them, investigating substance use disorder–gut microbiome interactions in humans and their roles in the development and progression of mental disorders is still a great challenge [185]. Similarly, it might be difficult to reproduce microbiome research data using animal models since many factors, such as co-housing with other animals, vendors, facility conditions, and other environmental conditions, give rise to differences in the composition and structure of the gut microbiome [186].

## 8. Conclusions

This literature review supports the idea that drug use is capable of influencing neuroplasticity, learning and memory, and reward circuit functions by targeting DNA methylation, post-translational histone modifications, and miRNA in different regions of the brain tissue. Moreover, these findings show that substance-induced psychosis can be associated with epigenetic alterations, and, hence, epigenetic-based therapies can be considered interesting approaches for alleviating psychotic symptoms in patients with SUDs. Dietary nutrients such as methyl donors (folic acid, vitamins B6 and B12, methionine, betaine, and choline) can serve as therapeutic agents for alleviating psychotic symptoms and depressive-like behaviors in patients with SUDs by reversing the epigenetic aberrations caused by substance use or modulation of the gut microbiome. Therefore, it is strongly reasonable to investigate new connections between them, since alterations in the human diet may be considered the easiest and first stage of treatment of substance-induced psychosis or may actualize a neuroprotective role in neuropsychiatric diseases.

## Figures and Tables

**Table 1:** Substance-induced DNA methylation alterations in brain tissue.

Type of Substance/Type of Study	Brain Region	Key Findings	Ref.
Methamphetamine/in male rats	Nucleus accumbens (NA) and dorsal striatum	Elevated expression of DNA (cytosine-5-)-methyltransferase 1 (DNMT1)	[52]
Methamphetamine/in male mice	Prefrontal cortex (PFC) and hippocampus	Alterations in the DNA methylation of several CpG sites of the *Arc* and the *Fos* in the PFC as well as *klf10* and the *Nr4a1* in the hippocampus of chronic methamphetamine-administrated mice	[53]
Methamphetamine/in male mice	PFC	Reduced levels of total histone 3 and 4 tail acetylation/elevated levels of DNA methylation	[54]
Methamphetamine/in male mice	Striatum	Demethylation within α-syn (SNCA) promoter	[55]
Methamphetamine/in male rats	NA	Elevated levels of DNA methylation of some K^+^ channel genes following methamphetamine self-administration	[56]
Methamphetamine/in male rats	PFC	Hypomethylation of BDNF-associated CpG islands	[57]
Methamphetamine/in human and male rats	PFC and hippocampus	Increased levels of BDNF methylation in human methamphetamine dependence and in the PFCs of methamphetamine-administered rats/decreased BDNF methylation in the hippocampi of methamphetamine-administered rats	[58]
Cocaine/in male mice	NA	DNA hypermethylation and elevated binding of MeCP2 at PP1c promoter/overexpression of DNMTs like DNMT3A and DNMT3B	[59]
Cocaine/in male rats	Caudate putamen	Elevated levels of DNA methylation at the PP1Cβ gene plus its binding to Mecp2	[60]
Cocaine/in honey bees	Central brain (excluding gnathal ganglia and optic lobes)	Changes in DNA methylation and, hence, derangements in consolidation of extinction memory	[61]
Cocaine/in male rats	NA/lateral habenula	Changes in methylation levels of *TAAR7B*, *PPP1CC*, and *A_2A_R* in high or low explorer rats	[62]
Cocaine/in male mice	Dorsal striatum	Hypomethylation in exon 3 of IRX2 in neuronal nuclei	[63]
Cocaine/in human	Striatum	Hypermethylation in a cluster of CpGs present in the body of tyrosine hydroxylase gene, including a putative EGR1-binding site	[64]
Cocaine/in human	Human PFC	The presence of differentially methylated regions relevant to genes involved in synaptic signaling and neuroplasticity	[65]
Nicotine or amphetamine/in male rats	mPFC, OFC, and nucleus accumbens	A reduction in global DNA methylation	[66]
Morphine/in male rats	Hippocampus	Hypermethylation of glucocorticoid receptor 1_7_ promoter after chronic morphine exposure and its association with hypothalamus–pituitary–adrenal axis dysfunction	[67]
Morphine/in male rats	Cerebellum/hippocampus/pons/medulla oblongata	Differential methylation of *IL1B* in the hippocampus, *Nr3c1* in the cerebellum, and *BDNF* and *Il6* in the pons after acute exposure to morphine/differential methylation of *NR3C1* in the hippocampus, *BDNF* and *COMT* in the pons, and *l1b* in the medulla oblongata after chronic exposure to morphine	[68]
Morphine/in male rats	Basolateral amygdala	Association between the DNA hypermethylation of *Gnas* and the reconsolidation of morphine reward memories	[69]
Heroin/in human	PFC	Detection of 1298 differentially methylated CpG sites (DMSs) between healthy individuals and heroin users	[70]
Heroin/in male rats	NA	Hypomethylation of the GABRD gene following heroin self-administration	[71]
oxycodone/in male rats	Hippocampus	DNA hypomethylation	[72]

**Table 2:** Substance-induced histone modifications in brain tissue.

Type of Substance/Type of Study	Brain Region	Key Findings	Ref.
Cocaine/in male rats	Striatum	H4 hyperacetylation at the cFos promoter after 30 min of a single injection of cocaine/H3 hyperacetylation at the *Cdk5* and *BDNF* promoters after chronic cocaine injection	[73]
Cocaine/in male rats	NA	Transcriptional activation of genes relevant to addiction by H3 acetylation	[74]
Cocaine/in male rats	Ventral tegmental area (VTA)	Association between elevated acetylation of histone 3 and cocaine-induced alterations in *BDNF* mRNA	[75]
Cocaine/in male rats	NA/hippocampus	Reducing expression-activity of class IIa HDACs in the NA following high-cocaine intake and reducing expression/activity in hippocampus following low-cocaine intake	[76]
Amphetamine/in mice	Striatum	Increased level of histone H4 acetylation after repeated treatment with amphetamine	[77]
Methamphetamine/in male rats	NA	Time-related elevations in acetylated H4K5 and H4K8/down-regulation of HDAC1 and up-regulation of HDAC2	[78]
Methamphetamine/in male rats	Striatum	Elevated acetylation levels of H4K5 and H4K8 in gene promoter regions	[79]
Methamphetamine/in male rats	Striatum	Reduction in HDAC6, 8, 9, 10 and 11 mRNA levels	[80]
Methamphetamine/in male mice	NA	Elevation of mRNA levels of HDAC3, HDAC4, HDAC7, HDAC8, and HDAC11 in HDAC2KO mice after methamphetamine injection	[81]
Methamphetamine/in male mice	PFC	Elevated acetylation status of histone 4 at class I HDAC1 and class IIb HDAC10 and reducing it at class IIa HDAC4 and HDAC5	[82]
Methamphetamine/in male rats	PFC	Hyper-acetylation of a number of genes (10 genes with H4 acetylation and 821 genes with H3 acetylation)	[83]
Heroin /in human	Striatum	Hyperacetylation of histone H3 at specific lysine residues (K27 and K23) at discrete genomic locations (GRIA1 and several genes involved in glutamatergic synaptic plasticity)	[84]
Heroin /in male rats	mPFC	Elevated levels of H3K9ac at the promoter region of brahma/SWI2-related gene-1 (BRG1)	[85]
Morphine/in male rats	Hippocampus	Enhanced binding of STAT3 to the CXCL12 gene promoter and elevating the acetylation of histone H4 in the CXCL12 gene promoter following repeated context exposure with morphine conditioning	[86]
Morphine/in male rats	Dorsomedial prefrontal cortex (dmPFC)	Enrichment of H3 acetylation at promoter regions of three genes (*Cdk5r1*, *Gabrb2*, and *Grm5*) in male animals	[87]
Morphine/in male rats	Hippocampus/basolateral amygdala	Association between morphine-withdrawal aversive memories and elevation of H4K5 acetylation and p-Brd4 activation	[88]
Oxycodone/in male rats	Dorsal striatum	Elevated levels of histone H3, phosphorylated at serine 10 and acetylated at lysine 14 (H3S10pK14Ac) in self-administered oxycodone animals using long-access paradigms	[89]

**Table 3:** Association between substance use and alterations in miRNAs in various brain regions.

Type of Substance/Type of Study	Brain Region	Key Findings	Ref.
Methamphetamine/in mice	Nucleus accumbens (NA)	Expression changes of 47 miRNA responsible for regulation of genes involved in modulation of metabolism, autophagy, and immune response	[90]
Methamphetamine/in rats	VTA	Alterations in the expression of 78 miRNA involved in addiction	[91]
Methamphetamine/in rats	NA	Overexpression of 166 miRNAs and down-regulation of 4 miRNAs following chronic treatment with methamphetamine	[92]
Methamphetamine/in rats	NA	Overexpression of 17 miRNAs and down-regulation of 23 miRNAs	[93]
Methamphetamine/in mice	NA	Elevated levels of miR-128, a miRNA responsible for regulating proteins involved in neuroplasticity, after repeated-intermittent methamphetamine use	[94]
Methamphetamine/in rats	Dorsal striatum	Reductions in the expression of miR-181a-5p and miR-181b-5p	[95]
Methamphetamine/in mice	NA	Down-regulation of novel-m009C expression, a novel microRNA involved in modulating methamphetamine-rewarding effects	[96]
Methamphetamine/in human (postmortem human brain tissue)	NA/VTA	Up-regulation of microRNA let-7b-3p in brain tissues of methamphetamine users	[50]
Cocaine/in rats	Dorsal striatum	Overexpression of miR-212 and, hence, regulating controlling the cocaine activity in brain reward circuitries	[97]
Cocaine/in rats	Dorsal striatum	Homeostatic interplays between miR-212 and MeCP2 for controlling the effects of cocaine on striatal *BDNF*	[98]
Cocaine/in rats	NA/striatum	Elevated expression of miR-212 and miR-137 in the dorsomedial and dorsolateral striatum, respectively, and miR-132, miR-137, miR-101b, and miR-212 in the NA shell	[99]
Cocaine/in mice	Striatum	Down-regulation of miR-124 by cocaine and thereby elevated levels of pro-inflammatory cytokines due to microglial activation in a TLR4-dependent mechanism	[100]
Cocaine/in mice	NA	Reduced levels of miR-124 following acute or chronic cocaine exposure	[101]
Cocaine/in mice	NA	Reductions of mmu-miR-34b-5p in vulnerable animals with high motivation for cocaine; reduction of mmu-miR-1249-3p in animals with high motor disinhibition	[102]
Heroin/in rats	NA	Mediating incubation of heroin craving by down-regulation of miR-181a	[103]
Heroin/in rats	Orbitofrontal cortex	Regulation of long-lasting heroin seeking by miR-485-5p	[104]
Morphine/in mice	NA	Alterations in the expression of 62 miRNAs	[105]
Morphine/in rats	Dentate gyrus	Overexpression of miR-132 following morphine treatment and its role in modulating the structural plasticity	[106]
Alcohol/in rats	Hippocampus	Overexpression of miR-3541, miR-125a-3p, and let-7a-5p and hypo-expression of their target genes (Nras, Prdm5, Suv39h1, Rnf152, Ptprz1, Apbb3, Mapk9, Ing4, Wt1, Nkx3-1, Dab2ip, Ripk1, Lin28a, and Acvr1c) in male alcohol-treated rats/decreased levels of miR-881-3p and miR-504, and overexpression of their target genes (Ube2g1, Naa50, Clock, Arih1, Cbfb, and Gng7) in female alcohol-treated rats	[107]
Alcohol/in human (postmortem brain tissues)	Hippocampus	Elevated levels of miR-34a and miR-34c in subjects with alcohol use disorder	[108]
Alcohol/in human (postmortem brain tissues)	Amygdala, NA, caudate nucleus, cerebellum, VTA, hippocampus, PFC, and putamen	Changes in the expression of 19 miRNAs	[109]
